# Notes on cost benefit of COVID‐19 lockdown

**DOI:** 10.1002/acm2.12970

**Published:** 2020-07-03

**Authors:** Michael Mills, Mary Beth Allen

**Affiliations:** ^1^ School of Public Health University of Louisville Louisville KY USA

The American population is without question suffering from fatigue. The stress of this ongoing pandemic has resulted in many Americans making the decision to return to normalcy as restrictions are easing in most states. In Kentucky as of early June, less than half of grocery and retail store patrons are complying with wearing face coverings in public. Retail outlets requiring face coverings are enduring some public resistance. Meanwhile, restaurants are allowed to reopen for in person dining and serve up to one‐third capacity and many houses of worship have been open for weeks. Furthermore, private gatherings for graduations, weddings, and holidays have become more common. Summer vacations and other settings with more lax restrictions have important implications for community‐level infection curves. Some Americans have also chosen to engage in rallies and social activism, which involves large crowd gatherings and travel between major cities. These expressions of fatigue coupled with re‐opening of economies are concerning as COVID‐19 case counts and hospitalizations have fallen mainly in heavily impacted settings like New York. https://abc7ny.com/health/coronavirus‐updates‐ny‐covid‐hospitalizations‐deaths‐continue‐drop/6248076/. https://www.washingtonpost.com/nation/2020/06/09/coronavirus‐live‐updates‐us/.

COVID‐19 is a novel virus that did not exist as a human pathogen until late 2019. With information continuing to accumulate, early clinical and management strategies do not reflect many current guidelines. Also, there is continued disagreement among experts regarding the future trajectory of the virus, with some predicting consistent infection and mortality numbers as others are sharing more hopeful predictions. The first reports of possible mutations and decreases in potency came from Dr. Alberto Zangrillo, the head of intensive care at the San Raffaele hospital in Milan in Lombardy. Zangrillo alerted Italian media of a study by his colleague that showed the virus was weakening and that, “in reality, from the clinical point of view, the virus no longer exists.” https://www.cnbc.com/2020/06/02/claim‐coronavirus‐no‐longer‐exists‐provokes‐controversy.html. These findings were echoed by researchers from the highly respected University of Pittsburgh Medical Center also showing that COVID‐19 at UPMC is declining in virulence and infection levels. https://triblive.com/local/regional/upmc‐doctors‐say‐covid‐19‐declining‐in‐virulence‐and‐infection‐levels/. However, World Health Organization experts and a range of other scientists have yet to find evidence to support any assertion that the coronavirus causing the COVID‐19 pandemic is losing potency. https://www.healthnewsreview.org/2020/06/reuters‐report‐is‐another‐classic‐case‐study‐in‐how‐not‐to‐cover‐covid‐19‐news/.

As the scientific evidence changes, public health policy and recommendations have also changed. The use of face coverings and masks is an excellent example as early advice from the WHO and the CDC does not represent current recommendations. The WHO initially maintained that only healthcare workers and the elderly at risk for infection should consider wearing a mask. However, updated recommendations include those who have difficulty maintaining social distance should wear a three‐layer cloth mask. https://www.webmd.com/lung/news/20200608/who‐changes‐stance‐says‐public‐should‐wear‐masks. Also, the CDC currently recommends the use of face coverings in public and continued social distancing, https://www.cdc.gov/coronavirus/2019‐ncov/downloads/cloth‐face‐coverings‐information.pdf, but these guidelines were updated from early advice discouraging the use of face coverings by the general population due to a shortage of personal protective equipment (PPE) for healthcare workers. https://www.nytimes.com/2020/03/31/health/cdc‐masks‐coronavirus.html These positions are in contrast to the prevailing advice in many nations. Japan, Korea, and China have reported very low Coronavirus infection and death rates despite limited and short‐term business and school closures, and even the lack thereof in some settings. Evidence suggests that low transmission and mortality rates can be attributed to cultural differences that support compliance of mask adoption. https://www.nytimes.com/2020/06/06/world/asia/japan‐coronavirus‐masks.html.

Social distancing policy and lockdowns enforced by the closure of nonessential businesses and mass gatherings remains a controversial policy throughout the US. With equivocal federal leadership, a wide variation in state policy, and a significant economic cost in all settings, much of the fatigue in the US is secondary to both short‐term economic measures and concerns of long‐term economic solvency. As of the second week in June, there is still no consensus of the actual cost of this policy in the US, as the number of net lives saved is also disputed. Ultimately, these are questions of great importance because of the enormous financial implications, including food and drug supply chains not to mention the financial hardships for many families and small businesses. In addition to current and future economic costs, there is no question that other important consequences resulted in routine medical and physical health as well as mental and social health costs. For cancer care specifically, important preventative screenings and elective surgeries were postponed. This may have prevented early detection and treatment in some cases and led to poor outcomes for many cancer patients. With other nonemergent care postponed, there is no question that common chronic illnesses were not diagnosed and treated timely and patients continue to delay care for many illnesses as healthcare systems resume most operations. As many Americans experienced fatigue from the pandemic as well as wrestled with financial concerns, social issues such as alcohol and substance abuse, domestic violence, and mental health risks including depression and suicide became exposed. https://www.realclearhealth.com/articles/2020/06/04/more_flexibility_still_needed_in_health_care_for_covid‐19_response_111052.html. https://thehill.com/opinion/healthcare/499394‐the‐covid‐19‐shutdown‐will‐cost‐americans‐millions‐of‐years‐of‐life. It is important to note that some of these impacts can be offset by a drastic decrease in gun violence, car accidents, and other crime that added unanticipated capacity to the healthcare system in some settings. https://abc7ny.com/nypd‐crime‐statistics‐new‐york‐city‐coronavirus‐nyc/6071137/ .

The failure of the federal government to act swiftly, which includes but is not limited to preparing the healthcare system for surges by expanding capacity and personnel, ensuring the availability of PPE, and dispatching test kits to all settings, left the US with few choices. Early identification and contact tracing were not an option and preserving healthcare resources including PPE was critical to prevent the healthcare system from collapsing. Given the available data and projections, this costly intervention was one of the few choices left in March 2020. The cost estimates of the lockdown are widely variable and based on varying assumptions. The Heritage Foundation reported that an 8‐week shutdown could raise the unemployment rate to as much as 23 percent and decrease immediate economic output by as much as $2 trillion. https://www.heritage.org/economic‐and‐property‐rights/report/the‐cost‐coronavirus‐shutdown‐orders. A report by the Congressional Budget Office projects that the U.S. gross domestic product will lose nearly $16 trillion over the next decade because of the pandemic. https://www.washingtonpost.com/nation/2020/06/01/coronavirus‐live‐updates‐us/. Terence Corcoran notes that the lockdown costs are certainly real at around $9 trillion. This includes the $2.5 trillion CARES Act passed by Congress and signed by President Donald J. Trump. https://business.financialpost.com/opinion/terence‐corcoran‐the‐price‐of‐life‐lockdown‐costs‐are‐real‐but‐are‐the‐benefits.

The mathematical‐level evaluation of this policy involves the calculation of cost aggregated to the estimates of lives saved as a result of the intervention while also considering the human cost of unintended consequences. This evaluation is standard in measuring the effectiveness of policy and in evaluating the need to continue ongoing programs. To determine the value of a life while also considering the economic costs of the lockdown from lost jobs and business closures requires the calculation of the value of a statistical life. The United States uses a value of $10 million. https://theconversation.com/the‐costs‐of‐the‐shutdown‐are‐overestimated‐theyre‐outweighed‐by‐its‐1‐trillion‐benefit‐138303. https://www.resourcesmag.org/resources‐radio/value‐statistical‐life‐and‐coronavirus‐alan‐krupnick/. https://papers.ssrn.com/sol3/papers.cfm?abstract_id=3379967.

In medicine, we use an analogous concept to specify the desired cost/benefit of a therapeutic or medical intervention. The benefit is measured in quality‐adjusted life‐years (QALY), and the valuation is usually $50 000 per QALY. https://www.ncbi.nlm.nih.gov/pmc/articles/PMC1497852/. https://www.tandfonline.com/doi/full/10.1586/14737167.8.2.165?src=recsys. https://www.science.gov/topicpages/q/quality‐adjusted+life‐years+qaly.

The QALY measure is a volatile number that depends on the nature of the intervention as well as the age of the patient. The older the patient, the fewer remaining life‐years. For example, if the majority of patients are over 65, then perhaps a reasonable valuation would equate to 20 quality‐adjusted life‐years, or $1 million. The order of magnitude difference between that and a statistical life reflects that the statistical life encompasses the entire economically productive life of the individual. For medical interventions most of the productive life for such individuals is in the past; note that 42% of COVID‐19 deaths are in nursing homes or assisted living facilities. This cohort represents just 0.6% of the US population. https://www.forbes.com/sites/theapothecary/2020/05/26/nursing‐homes‐assisted‐living‐facilities‐0‐6‐of‐the‐u‐s‐population‐43‐of‐u‐s‐covid‐19‐deaths/#7548521c74cd. This means that the statistical life estimate of $10 million might be an overestimate of the actual valuation of the lives lost, as these lives are mostly typical of the age range of the Medicare population and beyond their economically productive years. The true economic valuation could be closer to $1 million per statistical life than to $10 million.

The final question to consider is how many more deaths would have resulted if the US had opted for a more limited lockdown that was much less hazardous to the US economy. It is clear that anticontagion policies significantly slow the growth of COVID‐19, but what policies and how much slowing are optimal? https://www.nature.com/articles/s41586‐020‐2405‐7. https://www.nature.com/articles/s41586‐020‐2404‐8_reference.pdf. The best example of what might have happened is found in Sweden, and that country is an imperfect surrogate for the US economy. Sweden now has one of the highest COVID death rates in the world—almost 44 per 100,000 people. The number of U.S. deaths per 100,000 is just over 32. In Denmark, this number is 10, and in Norway, less than 5. https://www.bostonherald.com/2020/06/08/sweden‐backtracks‐on‐its‐low‐pain‐coronavirus‐plan/. Sweden’s plan is now considered to be less than optimal, but what if the US had followed it? How many additional net deaths would we have suffered due to COVID‐19? How many fewer deaths would we have suffered from postponing health interventions, suicide, and domestic violence, and alcoholism? Let us assume 100,000 additional deaths for the sake of a simple calculation. To save these 100,000 individuals, the cost to the economy was $9 trillion, or $90 million per statistical life. And keep in mind a large component of these individuals could have an economic life valuation much less than the $10 million standard value of a statistical life.

This calculation is obviously much simpler than the very sophisticated modeling used by the many government and academic institutions that struggle mightily to develop coherent and fair policy. Additionally, these numbers are offered in hindsight and are only rough estimates. Still, the result, that the US suffers an economic cost of $90 million per life saved, is quite sobering and merits reflection. It is important to note that policy makers enacted social distancing and lockdowns not only to save lives but also to save America’s fragile healthcare delivery system. The pause in many operations gave hospital systems time to prepare, create, often from thin air, testing systems, and enact critical policy that dictates how COVID‐19 patients are managed while also ensuring patient and healthcare worker safety. Lockdowns also allowed time to research treatment protocols, with some hopeful treatments proving ineffective and even dangerous while ramping up the supply for emerging treatments. Although this lockdown may have helped to save the healthcare system, it is clear that a second lockdown of the economy at a high cost to save a few lives is not a viable option.

As this pandemic remains ongoing despite fatigue from many Americans, future policy must be both evidence based and cost effective. Policy makers and health experts must be clear in messaging, consistency, and model the behavior publicly that we should all emulate. While some limited iteration of social distancing remains important, the single most critical intervention that all Americans should be encouraged to adopt is wearing a mask in public. This is a low cost and remarkably effective intervention that widespread scientific evidence continues to support. https://www.forbes.com/sites/alicegwalton/2020/06/13/face‐masks‐may‐be‐the‐key‐determinant‐of‐the‐covid‐19‐curve‐study‐suggests/?fbclid=IwAR2ZxIa4wJjSfEEfnnXVNToS‐CQOrW7x3Crb1V6WFFlQs6LHCMAjck8px‐c#72c1d9a56497 . There is some evidence that we may be able to be normal much sooner if we were all willing to do this very limited and simple thing. In fact, one study suggests that if more than 80% of the population are willing to participate, the results could be comparable to herd immunity achievable from population‐level vaccination. https://arxiv.org/pdf/2004.13553.pdf. If that is correct, a cotton face mask costing a few dollars could theoretically save millions of lives and billions of dollars.

